# An ethnographic investigation of medical students’ cultural competence development in clinical placements

**DOI:** 10.1007/s10459-022-10179-7

**Published:** 2022-11-12

**Authors:** Jia Liu, Shuangyu Li

**Affiliations:** 1 GKT School of Medical Education, Faculty of Life Sciences and Medicine, King’s College London, London, UK; 2 King’s Cultural Competency Unit, Faculty of Arts and Humanities, King’s College London, London, UK

**Keywords:** Clinical placement, Cultural competence, Ethnography, Healthcare education, Medical students

## Abstract

As a result of an increased understanding of culture’s impact on health and healthcare, cultural competence and diversity curricula have been incorporated into many medical programs. However, little is known about how students develop their cultural competence during their training. This ethnographic case study combined participant observation with interviews and focus group to understand students’ views and experiences in developing their cultural competence during clinical placements. The results show that students’ development of cultural competence is an individually varied process via four distinctive yet interrelated learning avenues. Immersion in a diverse healthcare environment contributes to students’ development of cultural awareness and knowledge. Observation of culturally appropriate or inappropriate practices allows students to enhance their practical skills and critical reflection. Interaction with other clinical professionals, patients, and their family members, enables students’ engagement within the busy clinical practice. Reflection helps students to actively think about culture’s impact on health and internalize the importance of cultural competence. Students’ learning via each avenue is interrelated and constantly interacting with their learning environment, which collectively contributes to their development. Integrating the results allowed the authors to generate a theoretical model that conceptualizes medical students’ cultural competence development in clinical placements, which unearths students’ cultural learning within the informal and hidden curriculum. This study provides a rare view of students’ development of cultural competence in clinical placements, which may inform the pedagogic development of cultural competence and diversity education in medicine and healthcare.

## Introduction

The inextricable link between culture and health has been widely recognized (Betancourt, 2003; Bhui et al., 2007; Napier, 2015). Culture plays a significant role in shaping individuals’ health-related views by determining their perceptions of diseases, experiences in consultations, ways of reporting symptoms, and adhering to treatment (Srivastava, 2006). The demand to understand health from a cultural perspective has become increasingly pertinent with the rise in globalization and the movement of people worldwide (Alizadeh & Chavan, 2016).

In this context, understanding and providing culturally competent care is seen as a strategy to create a healthcare system and workforce that can deliver accessible and effective healthcare for the whole population (Castillo & Guo, 2011). The World Health Organisation (WHO) called upon medical schools to “direct their education, research and service activities towards addressing the priority health concerns of the community, religion, and/or nation they have a mandate to serve” (Boelen et al., 1995, p. 3). Regulatory and accreditation bodies in many western countries stipulate medical graduates are required to provide culturally sensitive and appropriate care (Betancourt, 2003), such as the standards or graduate outcomes published by the Liaison Council on Medical Education in the United States (US) and Canada, the General Medical Council in the United Kingdom (UK), and the Australian Medical Council. In response, medical education at all levels has started to incorporate cultural competence education into its curriculum development.

### Cultural competence

The term “cultural competence” emerged in the 1980s intending to address the disparities in health outcomes between the white and black and ethnic minorities in North America. Cross (1989, p. 7) firstly used this term in medicine and defined it as “a set of congruent behaviors, attitudes, and policies that come together in a system, agency or among professionals and enables that system, agency or those professionals to work effectively in cross-cultural situations”. Cultural competence has dual emphases, *culture* and *competence*, so its definitions vary depending on which component is in focus (Shen, 2015). When focusing on *competenc*e, the attributes of the term are identified to include awareness, sensitivity, knowledge, and skill. When focusing on *culture*, it needs to be viewed from sociocultural dimensions such as race, ethnicity, religion, values, and health belief (Shen, 2015). Scholars (Campinha-Bacote, 1995; Doorenbos et al., 2005; Papadopoulos et al., 2004) have defined the term from different perspectives with emphases on certain elements of culturally competent care; however, a coherent and systematic understanding of the term is needed.

In addition to the difficulty in reaching a unified definition, the key attributes of cultural competence are disputed. Some of the key attributes described in the literature include *cultural awareness, cultural knowledge, cultural skill, cultural sensitivity, cultural interaction*, and *cultural understanding* (Balcazar et al., 2009; Burchum, 2002; Campinha-Bacote, 2002). In an earlier publication by the authors (Liu et al., 2021), we argue that the identified attributes reside in three interdependent domains—affective, cognitive, and behavioral. The affective domain of cultural competence embodies culturally appropriate attitudes, including respect, openness, and desire. It refers to the self-motivated willingness to explore new ideas and treat cultural differences positively and without judgment. The cognitive domain involves the development of cultural awareness, cultural knowledge, and understanding. This domain of development requires the consciousness of self-assessing one’s views, beliefs, and potential bias towards other cultures, as well as the acquisition of knowledge about other cultures and understanding its implications on health provision and behaviors. The behavioral domain refers to the pragmatic capabilities/skills to carry out effective communication and provide culturally appropriate healthcare to people from diverse backgrounds. Cultural skills, such as using respectful questioning, contextual inquiry, and appropriate non-verbal communication, are needed. So is the demand for self-reflection and critique of one’s thoughts, feelings, and behaviors.

### Cultural competence development

Although the importance of cultural competence has been acknowledged in health and medical education (Dogra et al., 2010, 2016; Dolhun et al., 2003), there are limited studies that have systematically explored the development of cultural competence among healthcare students. Garneau and Pepin (2015a, 2015b) define cultural competence development as a “complex know-act” that demands critical action and reflection, which healthcare professionals draw upon to provide culturally appropriate care in partnership with patients, their families, and communities, taking into account the political and social dimensions of care. They (Garneau & Pepin, 2015a) conducted a study to explore the development of cultural competence among nurses and undergraduate nursing students. The study concludes that individuals have to learn to establish a common ground that incorporates the culture of patients, the culture of themselves, and the culture of the care system, to overcome cultural barriers to effective care (Garneau & Pepin, 2015a). The barriers can be attributed to the different “beliefs and values” of the patient or clinician, “language spoken”, “professional standards” or “organizational structures and national policies” (Garneau & Pepin, 2015a, p. 1064). The importance of clinical placements in individuals’ development of cultural competence is highlighted as such experience provides learners opportunities to encounter diversity in ways that facilitate their learning.

Indeed, learning in clinical practice constitutes a vital component of medical education but is prone to variability. Previous research shows that students’ learning in clinical placements is not always structured and can be opportunistic according to a range of factors (Annear et al., 2016; Lofmark & Wikblad, 2001; Quilligan, 2015). Some of the facilitating factors include students taking reasonable levels of responsibility for independent learning, having sufficient opportunities to practice, receiving constructive feedback, and experiencing a supportive organizational culture (Lofmark & Wikblad, 2001; Quilligan, 2015). Obstructing factors include discontinued support from clinical supervisors, insufficient administration, a lack of opportunities to practice, as well as students’ perception of self-insufficiency and low self-reliance (Lofmark & Wikblad, 2001; Quilligan, 2015).

A comprehensive understanding of medical student learning encompasses the recognition that students learn not only in the formal classroom setting but also outside the classroom, which means the medical curricula can be categorized into three forms: formal, informal, and hidden (Doja et al., 2016; Hafferty & Franks, 1994; Karnieli-Miller et al., 2010; Lempp & Seale, 2004; Ozolins et al., 2008; Wear & Skillicorn, 2009). The formal curriculum represents the “actual course of study, the planned content, teaching, evaluation methods, syllabi, and other materials in any education setting from lecture halls to labs to seminar rooms” (Wear & Skillicorn, 2009, p. 452). The informal curriculum consists of the “ad hoc”, “unscripted” and “interpersonal forms of learning and teaching” taking place among and between faculty and students (Hafferty, 1998, p. 404). The hidden curriculum denotes students’ “opportunistic”, “unplanned”, and “idiosyncratic” learning in the clinical and institutional environment, such as through the physical environment of institutions or the interactions between students, academics, and clinicians (Wear & Skillicorn, 2009, p. 452). The informal and hidden curricula fall outside the formal curriculum but form a set of processes, pressures, and constraints in medical education. They have a significant role in nurturing students’ development of professional values and competencies because the institutional habitus of medical education does not take place in a “cultural vacuum” or within “a value-neutral environment” (Hafferty & Franks, 1994, p. 865). Instead, appreciable messages about professionalism, rightness and wrongs, and the identities of clinicians are embedded in the very structure of medical work and the unique learning environment (Hafferty, 1998). More recent research (Mountford-Zimdars et al., 2015; Webb et al., 2021) highlights that the informal and hidden curriculum has an impact on students’ sense of belonging, their self-image, and their interactions with teaching staff and peers, which play an important role in student academic performance.

However, limited research has explored the impact of the informal and hidden curriculum in medical education grounded in empirical evidence. There is a paucity of studies that have looked at medical students’ cultural learning in these curriculum settings, especially during clinical placements. How and to what extent the exposure to cultural diversity in clinical placements influences students’ development of cultural competence requires a systematic investigation. This study aimed to address this gap by exploring medical students’ views and personal experiences in developing their cultural competence in clinical placements. The results provide a rich ethnographic description of medical students’ experiences in the clinical environment and inform the pedagogical development of cultural competence education in medicine and other healthcare programs.

## Methods

Through the lens of constructivism, the authors have taken an ethnographic approach as it best suits our research aims. Constructivist researchers, who tend to resort to qualitative approaches, believe in multiple realities and argue that the understanding or meaning of phenomena is formed through participants and their subjective opinions (Creswell & Poth, 2016). The aim of ethnography lies in studying human behaviors and actions in social contexts and how these environmental contexts impose restraints on social interaction (Murchison, 2010). For this research, the investigation of students’ cultural competence development required a close and comprehensive examination of their views and experiences, which cannot be achieved by one single research method. Instead, ethnography combines different methods to gather rich and contextually detailed data via first-hand data collection (Murchison, 2010).

This study utilized participant observation, in-depth interviews, and focus groups with undergraduate medical students enrolled in a medical school in England. This is a two-stage study that aimed to provide an in-depth exploration of students’ views and experiences of their cultural competence development during their clinical placement. Stage One incorporated the whole cohort observation and shadowing of individual students. The observation was aimed to familiarize the ethnographer with the educational environment in the clinical context. Subsequently, shadowing was adopted to explore individualized students’ learning experiences. Stage Two incorporated the delivery of semi-structured interviews and a member-checking focus group. The interviews were conducted to elicit rich narratives of students about their views and experiences of cultural competence development in greater depth. In addition, one focus group was conducted for member checking after the preliminary findings of the observation and interviews were consolidated.

This research forms part of a larger study that explored medical students’ development of cultural competence in a range of educational settings. This paper focused on students’ development in their clinical placement contextualized in the UK National Health Service (NHS).^1^ Ethical approval was granted in November 2017 (LRS-17/18-5013). NHS R&D Approval (LOA854) was granted in April 2018 to allow the research to be conducted in the affiliated teaching hospital.

### Site selection and gaining access

This research was conducted in a London teaching hospital located in areas with socially, culturally, and linguistically diverse populations. The medical school has established a comprehensive cultural competence curriculum across its 5-year undergraduate MBBS program with an academic lead appointed to oversee its design and delivery (see Table 1). The teaching content is co-created by the members of a steering group on cultural competence, consisting of academics, researchers, clinicians, and medical students. In addition to bespoke lectures and workshops, clinical simulations using patient scenarios are used to teach cultural competence, integrating with the teaching of other subjects, such as clinical communication, ethics and law, professionalism, and interprofessional education. Most of these teaching subjects are encompassed in a Year-1 teaching block named “Human Values” (HV), under the Module “Introduction to Values Based Clinical Practice”. Students are assessed by their submitted writing reflective portfolio, where cultural competence is a key reflective point. Beyond Year 1, cultural competence is taught in themed workshops/webinars or relevant clinical blocks. In addition, cultural competence content also appears in the Single Best Answer (SBA) progress tests across all years. A cultural competence anchor statement is written as a general marking criterion in all the mark sheets for the Objective Structured Clinical Examination (OSCE) stations.

**Table 1:** Cultural competence curriculum at the medical school

A five-year MBBS* cultural competence curriculum
*Year 1*
Introduction to Cultural Competence lecture (1): This lecture introduces students to the rationale of developing clinical cultural competence, as well as related concepts (e.g., culture, diversity, equality, racism), and cultural competence models
Cultural competence & diversity workshop (1): This workshop encourages students to reflect on their own culture and its impact on the way they participate in social interactions in the healthcare context
Integrated patient scenarios (5): Cultural competence is listed as one key learning outcome along with the development of clinical communication, professionalism, medical ethics and law
*Year 2 and Year 3*
Living with disability workshop (1): This workshop helps students to understand the life of people living with rheumatoid arthritis and develop their cultural understanding and communication skills
Diversity workshops in Human Values Theme Week (3):
Interactive workshop on deaf awareness
Race/ethnic and equality in medicine and health
Medical interpreter and consultation Across language barrier
*Year 4 and Year 5*
Cultural competence webinar (5): This consists of a series of talks covering the topics of caring for homeless people, caring for LGBTQ + patients, working with interpreters, and understanding the intersectionality of patients who are homeless, substance users or sex workers
Race and health
The gender gap in pain management
Racial inequalities in women's health
Sexual assault
The role of the speak up guardian
Social determinants/structural competence (1): This lecture introduces students to structural cultural competence at the institutional and systemic levels

*MBBS* stands for Bachelor of Medicine, Bachelor of Surgery

Gaining access to the observation of students’ learning in the clinical setting was challenging as safeguarding patients is the primary goal in healthcare and any activity in the normal daily running of services is under scrutiny. This includes education and service delivery studies. In this research, communication with teaching staff in the HV teaching block paved the way for the authors to identify a relevant clinical block for participant observation and establish contacts with the administrative staff that was involved. JL was given access to observe students’ learning in the block Human Development, one of the four clinical blocks where the Year 2 medical students rotate 1 day a week for 7 weeks in total. In this block, students learn about the basic science applicable to women’s and children’s health, and how the scientific knowledge relates to clinical practice in obstetrics, gynecology, and pediatrics. The clinical block was considered relevant for observation as the provision of culturally appropriate care is listed as a learning outcome in the block introduction. The learning covers fertility, pregnancy, birth, growth, development, and adolescence. Students rotate in the Early Pregnancy Unit, the Gynecology Ward, the Antenatal Ward, the Antenatal Day Unit, the Postnatal Ward, and the Children’s Hospital. All students were given a briefing session by four clinicians in Week One. Each rotation day consisted of the morning ward rounds (around 9 am–1 pm) and an afternoon reflective workshop (2–4 pm) led by clinicians. The afternoon workshops were theme-based and led by clinicians who utilized different resources, such as videos and card games, to facilitate the discussion of a predefined theme relevant to human development. In the workshops, students took turns to present a patient case they had encountered in the morning ward rounds for discussion.

### Data collection

Data collection was carried out in two stages from April 2018 to April 2019. In Stage One, JL conducted 68 h’ observation over 1 month. In addition, five students were recruited for shadowing over the 5-week Human Development block rotation. All the students were in Year 2, and therefore, have completed the Year 1 cultural competence teaching and some of the Year 2 cultural competence teaching on campus. JL spent 1 day with each of the five students. A typical day started from around 9 am to around 4 pm. An observational journal (see “Appendix 1”) was created to capture information about the environment, activities, interactions, cultural elements, and JL’s reflections as an ethnographer.

In Stage Two of the study, 25 semi-structured individual interviews were conducted with participants recruited across different year groups. They have experienced different amounts of cultural competence teaching in the curriculum when recruited, with the Year 5 participants having studied in all cultural competence sessions. We used a semi-structured interview guide (see “Appendix 2”) which covered an extensive list of topics informed by a literature review. The amount of information students contributed to each of the questions varied significantly depending on the level of their clinical placement experience. Therefore, on average, each interview lasted between 20 and 30 min. All interviews took place either in the hospital or the medical school. In addition, a member-checking focus group (n = 5) was conducted at the end of the research. Participants in the shadowing and interviews were invited to participate. The focus group question guide (see “Appendix 3”) was informed by the preliminary results of the participant observation and interviews. Both the interviews and the focus group were audio-recorded and transcribed.

Participants for the two-stage research activities were recruited via different means including face-to-face recruitment, public advertisement, and snowballing. Table 2 summarises the recruitment and data collection. The combination of data collected from different methods provided a richer and more comprehensive understanding and interpretation of the phenomena in discussion.

**Table 2:** Participant recruitment and data collection

Stage one		*General observation*	*Shadowing*
	Data	68 h’ observation	25 h’ observation (n = 5)
	recruitment	n/a	Face-to-face recruitment in the Human Development block briefing session
Stage two		*Individual interviews*	*Member-checking focus group*
	Data	732 min’ recordings (n = 25)	106 min’ recording (n = 5)
	recruitment	Public advertisement; snowballing; invitation of the shadowed participants	Public advertisement; snowballing; invitation of all previous participants

### Data synthesis and interpretation

The authors employed an interpretative approach (Creswell & Poth, 2016), which involved iterative, inductive, and reflexive approaches to reviewing the fieldnotes, interview transcripts, and focus group transcript. JL followed five steps: (1) organizing the data; (2) reading and memoing the data; (3) describing the data; (4) analyzing the data; (5) interpreting and presenting the data. Regular meetings were held between the authors. SL randomly checked the documentation at all stages to ensure consistency. Ultimately, the data generated were integrated into individual ethnographic accounts and narratives. Fieldnotes were combined with accounts from interview/focus group participants to provide complementary details and explanations. Interview and focus group excerpts were edited for improved readability, during which incomprehensible or irrelevant texts were deleted and replaced with three dots in square brackets […]. All participants were anonymized. The demographic information of participants is provided in Table 3.

**Table 3:** Demographic information of participants (n = 35)

Demographic characteristics	Shadowed observation	Interview	Focus group
No. of participants	5	25	5
Gender	Male: 4	Male: 13	Male: 3
	Female: 1	Female: 12	Female: 2
Age	18–24 years old: 5	18–24 years old: 21 25–34 years old: 4	18–24 years old: 5
Year of study	Year 2: 5	Year 1: 6	Year 1: 1
		Year 2: 11	Year 2: 3
		Year 3: 1	Year 4: 1
		Year 4: 1	
		Year 5: 5	
		Intercalating: 1	
Sexual orientation	Heterosexual: 4	Heterosexual: 16	Heterosexual: 4
	Gay/lesbian: 1	Gay/lesbian: 3	Gay/lesbian: 1
		Bisexual: 3	
		Prefer not to say: 3	
Length staying in the UK	1–3 years: 2	1–3 years: 6	Less than 1 year: 1
	5–10 years: 1	3–5 years: 1	1–3 years: 1
	Since born: 2	5–10 years: 7	Over 10 years: 1
		Since born: 11	Since born: 2
Native language	English: 4	English: 20	English: 1
	Italian: 1	English and Arabic: 1	English and Urdu: 1
		Gujarati: 1	Finnish: 1
		Italian: 2	German: 1
		Mandarin: 1	South Korean: 1
Other languages except for English	Mandarin: 3	Arabic: 1	German: 2
	French: 2	Cantonese: 6	Swedish: 1
	Spanish: 2	Chadian: 1	
	Welsh: 1	French: 7	
	Cantonese: 1	Gujarati: 1	
		German: 2	
		Hindu: 2	
		Irish: 1	
		Malay: 1	
		Mandarin: 4	
		Portuguese: 1	
		Spanish: 6	
		Tamil: 1	
		Telugu: 1	
		Turkish: 1	
		Welsh: 2	
Ethnicity	White: 2	Arab (British): 1	Chinese (British) and other East Asian (British): 1
	Chinese (British) and other East Asian (British): 3	Black/African/Caribbean/Black British: 2	Indian (British) and Pakistani (British): 1
		Chinese (British) and other East Asian (British): 9	White: 3
		Indian (British) and Pakistani (British): 5	
		White: 7	
		Mixed/Multiple Ethnic Groups: 1	
Self-reported religion	Christianity: 1	Agnosticism: 1	Atheist: 1
	None: 4	Christianity: 5	Christianity: 1
		Hindu: 2	Muslim: 1
		Islam: 1	None: 2
		Jain: 1	
		Prefer not to say: 1	
		None: 14	
Self-reported social class	Upper middle class: 1	Upper middle class: 1	Middle class: 4
		Middle class: 17	Working class: 1
	Middle class: 3	Working class: 4	
	Prefer not to say: 1	Prefer not to say: 3	

### Reflexivity

Throughout the research, the authors maintained a reflective mind. Reflexivity prompted how our cultural backgrounds, professional backgrounds, experiences, and social identities shaped the research process.

JL’s non-clinical background caused several uncertainties at the beginning of the research. JL had the initial concern that her limited clinical knowledge might impair her understanding of students’ learning experiences, and subsequently affect the quality of the research. As an “outsider” (Murchison, 2010), JL may not be able to easily make sense of all the activities she saw in the hospital. Her presence may also change the dynamics of the teaching and care provision. Nevertheless, this anxiety was soon eased with JL’s growing understanding of the context through 12 months’ immersion in medical education prior to the ethnographic fieldwork. This significantly influenced her later observation and interpretation of data. JL’s identity as a PhD student back then not only allowed her to quickly build rapport with participants but also formed her insider’s perspective in this study of students’ experiences. It proved to be a positive approach and a flat platform to encourage students to open communication. Moreover, before commencing data collection in the hospital, JL sought advice from collaborating clinicians and academics. A clinical supervisor, who was also a university staff member, was assigned to JL. A comprehensive induction was put in place to help JL navigate through the clinical environment. Building a trusting relationship with clinicians, patients, and students was key. Once JL became an “insider”, the team no longer noticed her presence as an “intruder” to their practice. In return, JL also felt comfortable as part of the team. This positioning provided her with an opportunity to be neither an absolute observer nor an inclusive participant, but an “observant participant” with a pair of critical eyes (Murchison, 2010). It served the prime task of ethnographers well—to “get inside” the lives of a group of people and document their culture, views, and practices from an “outside” perspective.

JL’s cultural background could have influenced the way she interacted with potential participants. JL realized that it was easier to approach students who share common cultural elements, such as female students, students whose first language is not English, and students with cultural origins from Asia. This would have resulted in a disproportionate representation of participants from these backgrounds. This reflection informed the design of the recruitment which aimed to mitigate this potential shortcoming.

JL’s cultural background may have also impacted the data interpretation as it gave her a unique insight as a female Asian student herself. Such subjectivity strengthened the interpretation of experiences of participants who shared similar backgrounds. It allowed her to draw on the interpretation of others’ experiences as well as the reflections of the ethnographer’s own immersive experience (Murchison, 2010). JL “wallowed” in the data and constantly reflected from the views of both a participant and an observer (Murchison, 2010). This helped her to use the data analytical framework in a flexible manner, which allowed her to remain open to alterations, avoid overlaps, and consider previously unavailable and unobservable categories. Moreover, to minimize JL’s potential bias in data interpretation, SL and another supervisor EG (see Acknowledgment) cross-checked the coding scheme and held regular supervision meetings to discuss JL’s data interpretation.

SL’s role as the academic lead on cultural competence in the medical school has influenced the design of the project. The sites were selected based on SL’s professional network as well as the scope of the research. While the Human Development clinical block included in this research had more cultural competence content, other specialist blocks were not represented. This was mitigated through further data collection from the interviews and focus groups, but future research will benefit from expanding the scope of observations. SL’s insight into cultural competence constitutes the foundation of the research design, but at the same time, the authors were mindful of the potential imposition of his pre-existing knowledge upon the interpretation of data. Therefore, the authors regularly cross-checked a random selection of accounts across the data set and discussed different perspectives of interpretation and synthesis, which enhanced the consistency of the data interpretation. This would offset this limitation and maximize the use of SL’s expertise. The focus group run with the students allowed for further validation of the researchers’ interpretations. We were aware of SL’s identity as an academic and the power asymmetry it creates. Therefore, SL did not have direct contact with any student participants and only looked at anonymized data throughout the project.

### Trustworthiness

Trustworthiness in qualitative research refers to whether a study is credible, transferable, confirmable, and dependable (Connelly, 2016). The following procedures were undertaken to strengthen the trustworthiness of this research. A multi-method ethnographic design was adopted to increase the types of information and materials that were gathered, which allowed for multidimensional inquiry and triangulation across datasets. At the same time, such a design increased the types of students that were included in the research. Interviews, for instance, were a relatively non-invasive way to recruit a wide range of students, some of whom would not have participated in other means of data collection (e.g., observation). The focus group conducted at the end of this research served as a member-checking device, as JL shared the preliminary findings with medical students to obtain feedback. When reporting the results, the authors chose to present the “thick descriptions” (Geertz, 2008) of the observation in some key moments/events that involved detailed narratives explaining the situations and background context. A detailed description of key moments offered a nuanced understanding of students’ experiences by providing detailed, contextual, and multi-layered interpretations. In addition, JL discussed and debriefed the research progress regularly with SL and three academics with established expertise in this field. The feedback was discussed and incorporated throughout the research process. Transparency was demonstrated throughout the research process by documenting the procedures and decision-making in every stage of the research, which also enhanced its trustworthiness.

## Results

The results show that a variety of learning opportunities may contribute to students’ cultural competence in clinical placements. Students develop their cultural competence through four distinctive yet interrelated learning avenues: immersion, observation, interaction, and reflection. The sections below describe students’ views and experiences and elaborate on how cultural competence can be developed via the four learning avenues. The varied learning experiences that students have in the clinical environment are also highlighted.

### Learning by immersion: experiencing environmental diversity

**Fig. 1:**
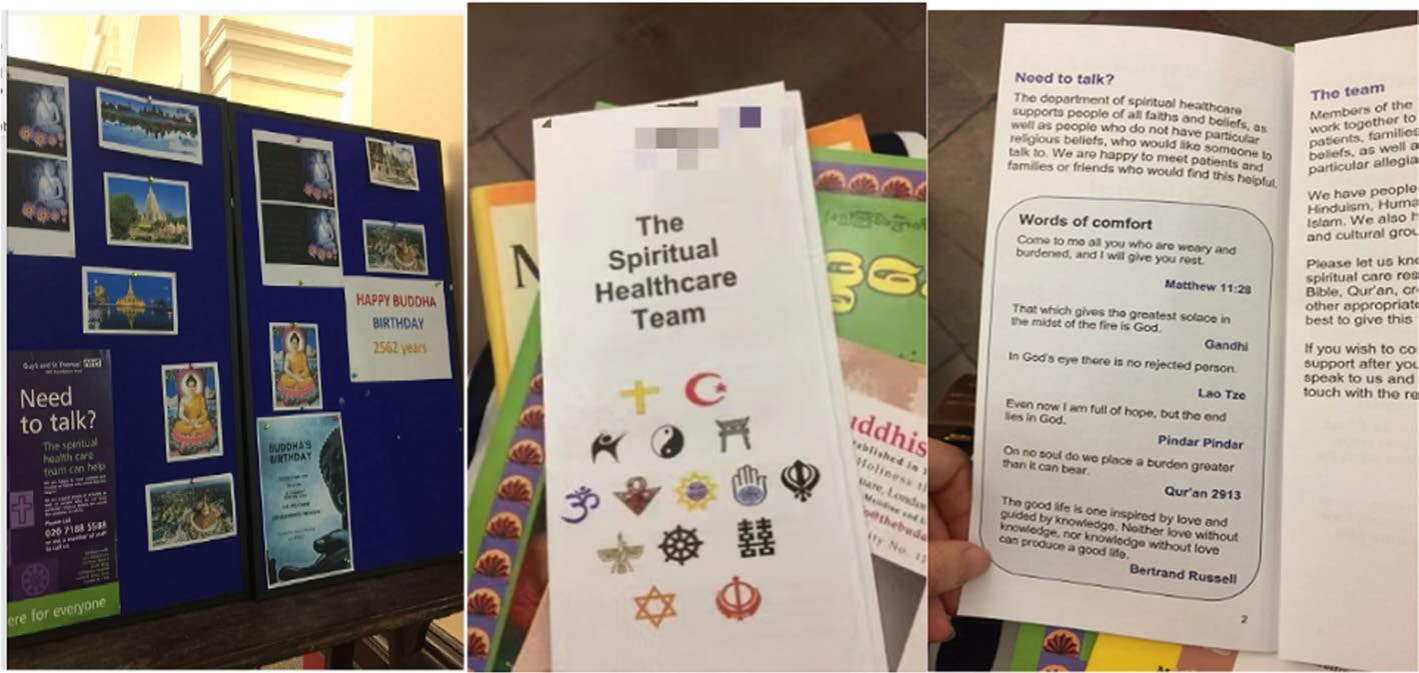
Multicultural artefacts in the hospital

Students develop their cultural competence in the clinical setting through their immersion in a culturally inclusive healthcare environment where multicultural artifacts can be seen. The hospital, where this research took place, presents multiple aspects of cultural diversity, which afforded students’ learning of cultural awareness and knowledge. Entering the hospital, one could see people of different genders, ages, ethnicities, and language backgrounds. Culture-related health information could be found on notice boards, posters, and leaflets (see Fig. 1).

Located in central London, the hospital is close to three boroughs, home to some of the most culturally diverse populations in England. 40% of the staff are from minority ethnic backgrounds. The hospital is named as a lesbian, gay, bisexual, transgender, queer and others (LGBTQ+) health leader by Stonewall, a charity that campaigns for LGBTQ+ equality. It has a diverse patient population that demonstrates multifaceted cultural differences (e.g., ethnicity, age, gender, sexuality, disability, religion, educational levels, and socioeconomic status). Bespoke services have been put in place to cater to the needs of minority patient groups. To better inform patients of these services, the healthcare staff runs regular public engagement events with local community leaders. On one of JL’s observation days, it was the Buddha’s 2562nd birthday. A celebration was organized on site.

JL’s fieldnotes: Next to the marble statue of Queen Victoria in the central hall of the hospital, a Buddhist event was taking place, celebrating Buddha’s 2,562nd birthday. Two monks in robes were giving a presentation about the life stories of Buddha, with constant reference to many of his quotes. By their side, an NHS staff member was distributing brochures covering content such as an introduction to the event, information about the Spiritual Healthcare Team, and the development of Buddhism as a religion. On the notice board among the different pictures of Buddha and the contact information of the NHS Spiritual Healthcare Team, three eye-catching words read “Happy Buddha’s Birthday”. Around 8–10 audiences of different ages and ethnicities were participating in the event.

The event served two purposes: religious event celebration and information giving. As the event was open to everyone, medical students were able to attend. Therefore, it offered them the opportunity to learn about Buddhism and the service provided by the Spiritual Healthcare Team, which they could not learn about from the formal curriculum. Immersion in such an environment, therefore, raises students’ cultural awareness and cultural knowledge. This resonates with the findings from the interviews and focus group, where students explained how they could acquire cultural knowledge and awareness about religious practices in clinical placements. According to Interview Participant 21:

[We are] exposed to quite a lot of different religious backgrounds in the hospital, like the Jehovah’s Witness. So before you even see the patients you can just talk to the medical staff, see what their [patients’] stances are, so then when you interact with the patient, you’re not just going in there completely blind, you have something to talk to and connect with them. And I think those are quite important knowledge to learn […] because we all have different cultures and different belief systems. […] So, when you’re talking to a patient you might be able to connect more easily.

Students mentioned that immersion in all forms of diversity can facilitate their understanding of cultural differences and improve their cultural awareness.

JL’s fieldnotes: Observation Participant 2 considered London an ideal place to study medicine as students in London are exposed to a diverse range of populations and diseases whereas medical students who study in relatively homogenous places normally do not have access. He described his first day of clinical placement in a London hospital as “being thrown into a wide spectrum of diversity”. Sitting in a small waiting room in the hospital, he could see staff from distinctively different cultural backgrounds working together. Patients of diverse ethnicities, language backgrounds, and dressing styles were waiting to be called. Many languages were constantly heard. Leaflets were translated into different languages and can be found in many public areas in the hospital, all demonstrating the “tremendous diversity” of the setting.

Students’ cultural encounters also challenge their existing values and attitude, offering new learning opportunities. Observation Participant 4 said that he used to think that he had a very high level of cultural competence thanks to his upbringing in a culturally diverse country before entering a UK medical school. He had taken a simplistic view to see cultural competence as just “remembering a list of dos and don’ts” of a particular ethnic group, which, for him, was not as important as developing other core clinical skills. However, this self-assessment was challenged when he entered the clinical placements, which then increased his commitment to acquiring further cultural knowledge.

JL’s fieldnotes: It seems that the participant considers himself quite culturally competent. He mentioned that growing up in a diverse place like Singapore has made him more culturally aware. Nevertheless, through immersion, he has developed a renewed understanding of culture and diversity. He said that he did not know there exist so many choices for gender and sexuality before coming to London, demonstrating that he has acquired more cultural knowledge in this respect.

Although the observation was conducted in a London teaching hospital, most students acknowledged that cultural diversity is not limited to big cities but exists anywhere. This is because cultural diversity is not only rooted in visible sociocultural factors (e.g., race, ethnicity) but also determined by less visible individual factors such as one’s sexuality, education, and socioeconomic status.

### Learning by observation: seeing and modeling

Observation involves seeing not only how healthcare staff approach cultural issues, but also their interactions with patients, family members, and other staff members. Observing how different patients view sensitive health issues and communicate their symptoms is beneficial for students to enhance their cultural sensitivity and develop respectful behaviors. Interview Participant 17 said that the observation showed her the nuances of how patients view sensitive issues across cultures:

When I was in GP^2^ [General Practice], one of our patients […] was Somali and she felt so uncomfortable. […] It was clear by her symptoms that she had UTI [Urinary Tract Infections], but she was so uncomfortable talking about it or even discussing it with the nurse, who was a female, and I was female in the room. […] She was still very uncomfortable, so I guess it’s important to be just aware of the culture. […] So, we can be a little bit more sensitive than we usually are to the differences, to the nuances of different cultures.

Observing culturally appropriate practices improves students’ cultural awareness and motivates them to acquire effective cultural skills. When shadowing Participant 2 in the Early Pregnancy Unit, JL found that observing a senior nurse conducting the triage consultations with patients of diverse cultural backgrounds (e.g., ethnicity, language background) in a culturally appropriate manner helped the student enhance his strategies of communicating with patients. The student expressed that observing the senior nurses’ non-judgmental and empathetic approach to diverse patients has contributed to his development of cultural openness and cultural awareness. JL also noticed that the student, in his subsequent interactions with other patients, incorporated similar linguistic terms and communication styles, such as using body language or checking the understanding of non-English speaking patients. Similarly, Interview Participant 24 explained how he has benefited from learning by observation:

Observing the interactions different people have within clinical placements, and not just observing patients themselves individually and clinicians […] and all those things, but interactions between these two groups of people would probably help you develop [your cultural competence]. Because you know these [experienced] healthcare professionals, they have seen these patients constantly, and they might have this instinctive way of reacting to things or replying to their patients that we haven't developed. […] So, seeing those nuances in their conversations might help us pick up something […] in their body language, the way they say things, even like oblique questions, what kind of questions they use, the tactics.

Although cultural competence can be enhanced through students’ general observation, students are more motivated to learn if they have observed a role model who demonstrates cultural competence. Interview Participant 14 explained how identifying her General Practitioner (GP) as her role model encouraged her to reflect on her attitudes toward culturally diverse patients:

My GP is kind of my role model. I think he knows the fine line between that being prejudiced and not using stereotypes to shape how you treat your patients. […] We had the experience that the GP has to explain to a patient why she didn’t need antibiotics to treat her viral infection. The patient, (a) she didn’t [speak] English very well, and (b) I don’t think she was very well educated, and (c) she expected a pill […] to work on whatever illness you have. And that’s what she wanted. Then my GP spent a lot of time trying to explain these terms the patient doesn’t understand. […] He would not get too frustrated that she didn’t understand it, […] but explained it to the patient in a way that we want you to feel as comfortable as possible. Because you have to take account of people’s knowledge is very shaped by their own culture as well. […] That’s the kind of doctor I want to be.

On the contrary, observing culturally incompetent behaviors helps students to learn as these incidents provide opportunities for students to recognize cultural inappropriate practices that may be discussed in campus-based teaching. Observations of such nature enable students to become more aware of the negative impact of culturally inappropriate care on patient outcomes. In this research, three participants mentioned that they have witnessed cases that dealt with cultural inappropriateness, which has led to their critical reflection on cultural competence. The three cases were about ineffective communication between a clinician and a patient believing in alternative medicine, a dangerous pregnancy delivery due to linguistic challenges, and a doctor’s stereotypical assumptions of dietary preferences based on patients’ ethnicities.

### Learning by interaction: gaining hands-on experience

Interacting with patients and their family members allows students opportunities to explore patients’ cultures in great depth and to rectify potential oversights caused by linguistic and cultural challenges. This type of interaction contributes to students’ acquisition of knowledge and the improvement of skills in resolving cultural barriers, caused by differences such as language, religion, diet, ethnicity, health beliefs, gender, sexuality, and family cultures. For example, Interview Participant 1 gave an example of how interacting with a patient unexpectedly motivated him to explore the connection between a patient’s cultural background and his diet:

I was going to this patient with a rheumatoid [condition] with the pain in the knees and […] chat about the history. We [with another medical student] asked about the diet and then he said, “oh it’s African food”. It wasn’t, it wasn’t the main point of what I was trying to find, but in those cases, you get a passive sparkle, and you have to actively decide to “oh let me investigate that a bit more” because I’m curious, I want to know a bit more about the impact of diet [on his condition]. So I asked him to tell me more […]

Multilingualism among participating students in this research was not uncommon. Quite often the opportunity arose for them to utilize their multilingual skills in a clinical interaction with patients, which often contributed to more satisfactory patient outcomes. In this study, speaking the same language with non-English patients facilitated students’ communication with patients because it helped to reduce linguistic barriers. Students’ interactions with these patients also foregrounded the challenges that patients face when seeking care and made the students more empathic toward these patients. Such experience increased some students’ cultural humility. They realized that the scope of the challenges these patients face is so profound that being able to speak the patient’s language is far from enough to provide equitable care to them. This is evident from Observation Participant 5:

I was thinking if you [the researcher] were here last week, we had so many cases happening. For example, one patient came to the Postnatal Ward herself and she does not speak English. They had an interpreter booked via Language Line, but that interpreter was very late. What’s worse, the clinicians and nurses were not aware that they were expecting an interpreter. As the lady was about to deliver, the staff asked around who can speak Spanish. Lizzy [my clinical partner, pseudonym], said she can speak Spanish and tried to help. Unfortunately, it turned out that medical English is not as easy [to translate] as a daily conversation. At last, they had to use [Google Translate] to help the lady deliver. The delivery went OK. But the team was so nervous as being unable to communicate in the same language in a critical medical moment can be very daunting.

Although the diverse student body increases workforce diversity, there is a “flip” side to this. During the observation, JL noticed that patients from minority ethnic backgrounds were likely to seek help from staff and students with similar ethnic backgrounds. While multilingual and multicultural students add to the diversity of the healthcare team, which is often welcomed by the patients, some students considered it a “burden” to themselves as it distracted them from their training. According to Focus Group Participant 5, a British Pakistani who has lived in East London since he was born:

A lot of patients think I’m Pakistani Muslim, fair, and a lot of people think I’m Hindu-Indian, and patients would be super friendly. They would come out to you and just start speaking Urdu or Hindi […] So, I would speak to them, and they would want to just stick with you and just talk, even more, they’ll tell you what’s going on at home and personal things as well. Sometimes you’re busy like “I really have to go”. [But] they’re like “oh you can understand obviously”. I think just [because] my ethnicity, and my last name is […], so they’ll be so friendly to you, if you’re like the same ethnicity. […] They will say anything.

### Learning by reflection: achieving deep learning

Reflection helps students to consolidate and internalize their learning based on what they have seen or been taught. The afternoon workshops, as part of students’ placement learning, provide a chance for students to discuss their encountered patient cases and reflect within a safe environment. Although the workshops primarily focused on medical perspectives, topics reflected varied students’ experiences and interests. Cultural issues may be discussed if relevant. For example, in a workshop that focused on children’s development, the cultural knowledge that young children growing up in bilingual or multilingual environments may experience speech delay was discussed with reflective cases provided by clinicians and students.

In addition to the structured reflective sessions, students mentioned in the interviews that they had the opportunity to reflect on their clinical experience in their writing assignments (e.g., reflective essays, portfolio, artwork) as part of other modules in their formal curriculum teaching, such as Year 2 Longitudinal Placement: General Practice, or Year 5 Global Health and Elective. These assignments do not focus on cultural competence, but students’ unconscious learning may surface if cultural topics were chosen. When working on the assignments, students expressed that they may actively reflect on their clinical experience about the cultural issues they encountered and self-reflect on their cultural competence, which process can contribute to their improved understanding and recognition of cultural competence. Some students allowed JL to read the reflective pieces they submitted for the Year 2 Clinical Humanities Project. These reflective pieces showed that some students were able to bridge their clinical practice with the cultural competence theories and concepts they have been taught in classroom teaching.

JL’s fieldnotes: Another way for students to develop their cultural competence is by writing reflective portfolios/pieces as part of their formal curriculum assessment. As the medical school uses reflective writing to assess students’ learning in value-based medicine (among which cultural competence is a learning outcome), students mentioned in the interviews that the reflective assignment submitted for other modules in the formal curriculum enabled their deep thinking about their placement learning, both positive and negative, and encouraged them to reflect on a range of issues including clinical communication, professionalism, and cultural competence.

Cultural competence learning opportunities may implicitly take place, with some students not realizing their learning or reflecting on the influence at a later stage. The focus group participants agreed that with so many targets to achieve in clinical placements, the importance of developing cultural competence is sometimes underestimated or neglected. However, they unanimously agreed that through constant clinical exposure, they have enhanced their cultural competence when reflection is triggered at a later stage. According to Focus Group Participant 3:

I think you may not feel like you are learning, much of cultural competence, but then when you compare yourself to how you were at the beginning of the year, you realize how much you’ve learned. […] You realize how, how you develop your cultural competence.

### Varied learning experience: the impact of the informal and hidden curriculum

Students develop their cultural competence through engaging with patients, clinicians, themselves, and within the wider healthcare environment. No single student has the same experience as the other. In this research, the authors found that students had varied learning experiences with differences in the length of their placements, the support they received, and the number of patients they saw. The results helped us to identify the contextual elements that are central to constructing students’ cultural learning environment in the clinical setting, which may be shaped by their personal trajectories, available opportunities to practice, institutional environment, and cultural competence teaching in the formal curriculum.

Students’ individual trajectories have an impact on shaping their cultural attitudes, knowledge and understanding, influenced by factors such as their personalities, self-confidence, ethnic and linguistic backgrounds. For example, JL observed that students with ethnic minority backgrounds were more likely to be approached by patients from a similar cultural background. Students growing up in a culturally diverse region tended to have a higher level of self-confidence in cultural competence. Personality was also mentioned by participants as an influencing factor as more outgoing and confident students were more likely to connect with patients. According to Observation Participant 5:

Sometimes the biggest barrier is the patient not being happy with a medical student seeing them, which you can’t really help. […] The other one for me is that especially you know just me as a person, […] I’m a bit timid, not quite too keen on asking the patients for something if I’m not pushed for half the time.

Whether students have sufficient opportunities to engage in cultural encounters rested on their “luck”. “Luck” was referred to by some students as a key determinant of whether they have sufficient opportunities to practice. According to Observation Participant 1, his rotation at the hospital was generally well-organized but sometimes depended on “luck”.

JL’s fieldnotes: Participant 1 was scheduled to practice at the Children’s Hospital to explore the theme “Growth”. After observing the senior nurse conducting measurements by using a machine, he wished to practice the skills but was not lucky enough to do so on this occasion. There were surprisingly few patients that day. After waiting for one hour and a half and seeing no sign of any patients, the participant decided to call it a day at around 10:40 am. He also mentioned that students’ experience varies significantly depending on lots of unpredictable factors; however, there are no detailed guidelines for them to follow. He deemed that students’ learning experience is also attributed to their initiatives and judgment to utilize opportunities. As for this day, he decided to finish his placement early as he thought it would be more productive to use the time to prepare for his coming examinations rather than waiting there with nothing to do.

Apart from luck, the institutional culture of the healthcare organization has a key role in constructing students’ unique cultural learning environment. Participants expressed that they experienced varying levels of support when rotating in different clinical sites. Whilst some sites have a more established culture of providing continuous student support from both the supervision staff and the clinical team, other organizations have relatively “loose” management leaving students to mostly “take their own initiatives”. Moreover, the institutional culture of a healthcare organization partly depends on the nature of the care environment. Some interview participants expressed that rotation in the primary care setting, such as the General Practice, could benefit their cultural knowledge markedly compared to rotation in the hospital. This is because learning in the primary care setting is generally more organized and allows them more chances to observe experienced clinicians and interact with patients. The demographic feature of patients registered in a GP clinic also helped students to develop their cultural competence in working with patients from certain cultural backgrounds. According to Interview Participant 8:

Actually, I would say that kind of cultural thing probably comes up more in GP, because you deal with a lot of social issues particularly in the GP. They have time, and often, you know, multiple members of the same family will be registered at that GP practice, so the GP I imagine over time would have to deal with the culture that is within that family, and that culture may be very different from the GP’s own culture.

In addition, the cultural competence teaching that students experience in the formal curriculum has an impact on students’ learning in the clinical setting. In this study, students who received systematic teaching on cultural competence were more likely to express openness to cultural diversity and demonstrate awareness of their unconscious bias. They were also more capable to identify culturally competent/inappropriate practices within the overall culture of medical practice and reflect on their experiences. In turn, students’ participation in the clinical setting enabled them to link their classroom training with practice, creating further learning opportunities. Three participants expressed that the campus-based teaching “flagged” the importance of cultural competence for them, making them “more aware” that this is a key competence for practicing clinicians. However, that exposure to cultural encounters does not always lead to cultural competence development if the students are not ready. A lack of incentives/awareness in developing the so-called “soft clinical skills”, including cultural competence, minimizes students’ learning. Interview Participant 11 expressed:

In our placements, this just involves us talking with patients most of the time. I have seen patients from different places, different cultures, but when I talk to them, it’s a lot about their personal life and their condition. They rarely go explicitly to their culture and stuff, so I guess the difference is not something that comes in front of my brain when I talk about it. […] I do acknowledge there are cultural differences, but it’s not something [that] is imperative to me, or to the scenario.

### A cultural competence development model

**Fig. 2:**
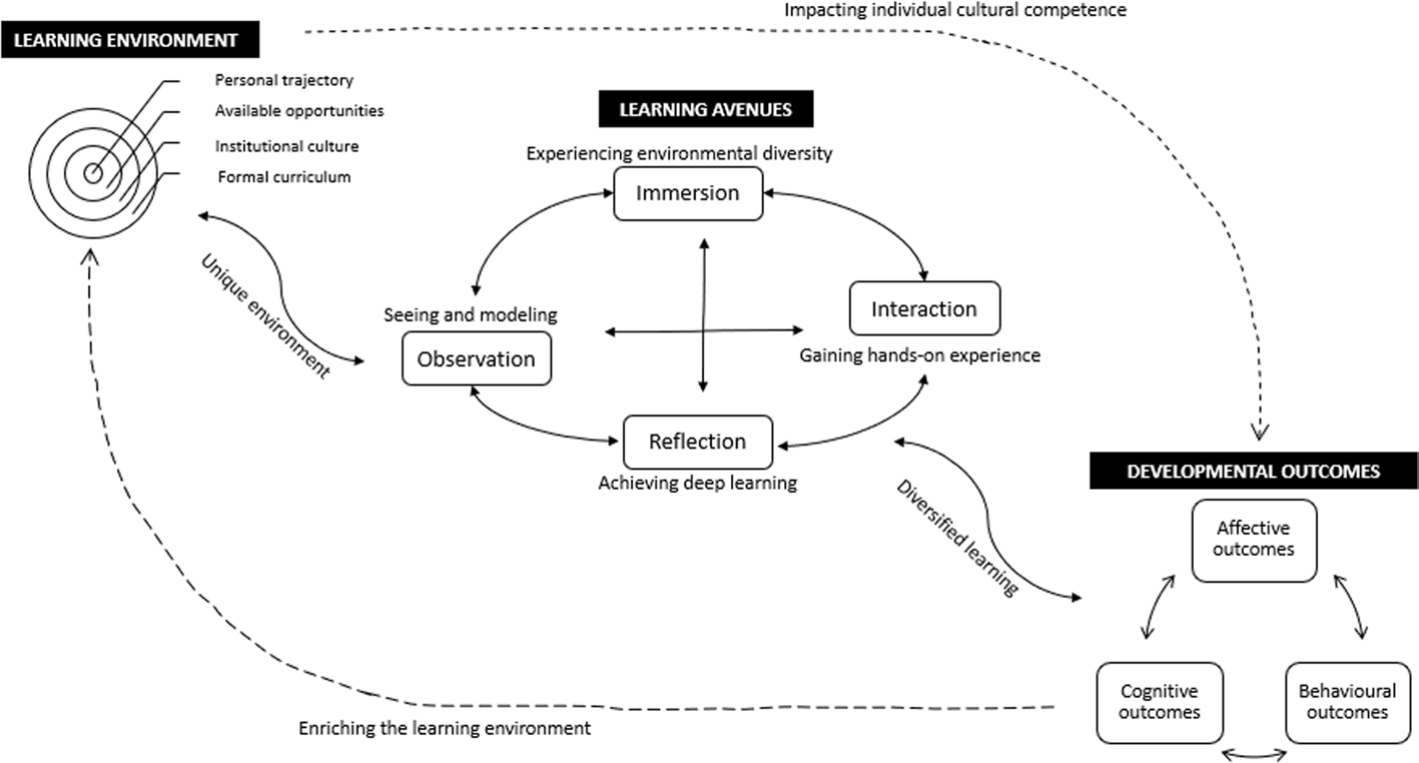
Students’ cultural competencedevelopment model in clinical placement

The ethnographic study enabled the authors to formulate a new model to describe the process through which students develop their cultural competence in their clinical placements (see Fig. 2). Students’ *learning environment* sets the baseline for their development of cultural competence, which pre-exists before further development takes place in clinical placements. Students have varied starting points depending on (1) their personal trajectories, (2) the learning opportunities they had before and the opportunities they may obtain in the clinical setting, (3) the institutional culture of the medical school and the affiliated clinical sites, and (4) the medical curriculum they are provided with. Apart from (4), other elements can vary drastically, putting students at different starting lines to further develop their cultural competence in clinical placements.

An influential personal trait identified in this research is students’ levels of interest and sensitivity in engaging with cultural encounters. This is influenced by each student’s upbringing, early educational background, and personality. For some participants, being an international student, or speaking a foreign language has made them more aware of the diverse cultural beliefs and the impact of communication on interpersonal relationships. A student’s personality also plays a significant role as more outspoken and extrovert students tend to have more encounters with patients, clinicians, and peers, and they are more active in reflective discussions. This, to some extent, affects whether students have sufficient opportunities to practice. In addition, the availability of opportunities also depends on students’ “luck” due to the opportunistic and idiosyncratic nature of the clinical setting. The institutional culture of the hospital where the placements are based is essential as it largely decides the level and means of support provided to students, which adds to the varied learning amongst students. Another environmental element that affects students’ cultural competence development is their formal curriculum. With more teaching on cultural competence in the formal curriculum, students are better prepared for their placement learning where they learn to put their classroom-based learning into practice.

The middle section of the diagram indicates the four *learning avenues* for students to further develop their cultural competence in clinical placements. The four learning avenues are not standalone entities but interact with one another and collectively contribute to students’ development of cultural competence. Immersion in a culturally diverse healthcare environment contributes to students’ development of cultural awareness and knowledge, which may motivate students to learn in the other three avenues. Observation allows students to enhance their practical skills via interacting with clinicians and patients and critically reflect on their cultural competence and that of clinicians. This may consequently create learning opportunities for both students and clinicians. Interaction with other clinicians, patients, and their family members, enables students’ engagement within the real-time care provision. Interactional learning sometimes takes place simultaneously with observation and can trigger students’ deep learning when reflected upon at a later stage. Reflection helps students actively think about culture’s impact on health and internalize the significance of cultural competence. In turn, the active and deep learning via reflection contributes to students’ enhanced motivations for cultural learning via other avenues. While each avenue is distinctive from one another, together they are complementary and allow for students’ diverse and dynamic learning and development.

The *cultural competence developmental outcomes* listed on the right side of the model show that medical students, while contextualized in their unique learning environments, can take advantage of the four learning avenues to gradually achieve cultural competence outcomes in the affective, cognitive, and behavioral domains. As discussed in the Introduction, cultural competence attributes are categorized into the three interactive domains, which collectively sum up the requirements that are essential to the provision of culturally appropriate care (see our paper, Liu et al., 2021). We argue that the three domains are interdependent. Individuals’ cognitive development in cultural awareness, knowledge, and understanding, contributes to the development of the “affective competencies” in openness, humility, sensitivity, and willingness to self-empower. The affective competencies reciprocally benefit healthcare professionals’ continuous acquisition of cultural knowledge, which may lead to improved awareness and understanding. Development in both domains will add to the behavioral development that requires effective cultural skills and clinical communication. Meanwhile, self-reflective professionals continue to evaluate their skills and practice, which in turn will enhance their affect and cognition.

## Discussion

Medical students’ cultural competence development in clinical placements is a complex process where the learning can be opportunistic, unplanned, or as part of formal teaching. The results of this ethnographic research contribute to the theoretical understanding of this process with the proposition of a Cultural Competence Development Model. We argue that students’ cultural competence development is a dynamic and interactive process, in which students are constantly influenced by distinctive learning environments. The clinical placements offer students new learning opportunities via the four identified learning avenues, through which students develop their cultural competence in the affective, cognitive, and behavioral domains respectively. The three-domain categorization, which is in line with previous literature (Liu et al., 2021), specifies the outcomes that future doctors need to achieve, providing a high-level structure for educational development. Moreover, individual cultural competence may result in positive changes in the institution and the wider learning environment, which in return expands the learning opportunities for students.

Our model (see Fig. 2) highlights that each student has a unique learning environment, which may foster their development of cultural competence. Students bring their personal trajectory and cultural competence learning in the formal curriculum when entering their clinical setting. This means that students interact with the institutional culture of a healthcare organization differently, resulting in their varied opportunities in engaging in cultural encounters. Students’ personal trajectory is influenced by all their life experiences gained from, for instance, their upbringing, early educational background, and lifestyles. Those with more experience may add value to other students’ learning experiences. The discussion of the unique learning environment is in line with previous literature that delineates the contextual elements that foster healthcare students’ development of cultural competence, including one’s personal trajectory and the social/political dimensions of care (Garneau & Pepin, 2015a). However, in addition to recognizing the influences of individual learners and the wider clinical environment, our model acknowledges the role that formal curriculum plays in constructing the cultural learning environment for medical students, highlighting the interconnected relationship of the formal, informal and hidden curriculum.

The biggest contribution of this research is that it identifies four distinctive and yet interrelated avenues whereby students develop their cultural competence in clinical placement. Immersion exposes students to unique encounters with individuals of other cultures as well as multicultural artifacts presented within the clinical setting. Students may improve awareness and knowledge of the diverse practices among patients and the clinical team. Previous studies (Canfield et al., 2009; Crampton et al., 2003; Larson et al., 2010) show that immersion in multilingual and multicultural environments is an effective means to learn about not only people of other cultures but also the culture of oneself. This research also shows that cultural immersion is omnipresent. However, learning is only possible when both students and educators can see culture beyond race and ethnicity, but rather as an identity formed by the individual’s multifaceted sociocultural determinants. This finding is consistent with the argument held by Canfield and colleagues, who state that “most communities have cultural diversity, at least to some extent” (2009, p. 320).

Observation is a crucial means of learning in clinical placements. Students intend to model their practices on that of culturally competent clinicians. This is consistent with the effects of role modeling, when the senior members of the community enact through their behaviors, both tacitly and explicitly, how problems of the discipline are approached, how colleagues are regarded, and how knowledge is formed and used (Benbassat, 2014; Murray & Main, 2005). Students benefit significantly from observing senior clinicians who demonstrate sensitivity to cultural barriers, have keen attitudes to acquire cultural knowledge and possess effective interpersonal skills to build compassionate relationships with patients. Seeing the opposite; however, may reinforce negative cultural stereotypes and trigger malpractice that may lead to compromised patient care. Nevertheless, our research indicates that formal curriculum teaching on cultural competence can equip students with the awareness to identify inappropriate practices if such practice has been discussed. Sometimes, students are confident enough to bring such observations into the reflective sessions with the clinicians, which forms a mutually beneficial professional developmental opportunity for other students and clinicians (Ironside, 2005).

Interactional learning couples closely with learning through observation. Students’ active engagement and interaction within the people environment bridge the gap between theories or concepts they learn in the formal curriculum and the real clinical practice, which requires life-long learning. This offers a testing ground for their attitudes and critical thinking, which fuels their ability to make situational judgments and act culturally appropriately. The process, in return, highlights the impact of their own unconscious bias on their actions and how that may hinder care for patients. The real-time pressure on their communication and clinical decision-making also foregrounds their ability gaps to be addressed in their future learning.

Throughout the observation or interaction process, “reflection-in-action” (Schön, 1987, 1995) may occur during a cultural event. The real-time pressure and decision-making within a clinical cultural encounter may help students to reframe/rework the problem from different perspectives, establish where the challenges fit into their learning, and understand the elements and implications present in the problem, its solution, and consequences. This is pertinent to one’s cultural competence development because reflective skills are essential for developing an understanding and appreciation of each other’s values, understanding, and unique behaviors (Almutairi et al., 2015; Olson et al., 2016). This study shows that if organized reflective sessions provide students with a safe environment for deep learning, they can trigger students’ critical reflection on their involved cultural encounters in the clinical setting. So are the reflective writing assignments required by relevant modules in the formal curriculum. The way the reflective activities/assignment is organized determines whether cultural practices can be picked up and discussed in detail. If well organized, these activities are likely to increase the likelihood for students to have “reflection-on-action” (Schön, 1987, 1995) after the occurrence of a cultural event. Reflection-on-action allows students to think back on what has happened in the situation to determine what may have contributed to the unexpected, and what has been learned from this situation may affect future practice.

The four interconnected learning avenues are consistent with the *experiential learning theory* (Kolb, 2014), which consists of four phases that underline individuals’ learning cycle: concrete experience, reflective observation, abstract conceptualization, and active experimentation. Kolb’s (2014) theory describes a holistic approach to individuals’ learning, which discusses the key components of “learning-by-doing”. Among the four learning avenues identified in this study, interactional learning with clinicians, patients/families, and immersion in the wider clinical environment allow students to gather “concrete experience” in working with cultural differences. Observational learning through witnessing culturally (in)appropriate practices helps students to view and analyze their experienced cultural encounters from multiple perspectives, which contributes to their “reflective observation”. Reflecting on these experiences either as a group or individually further lead to their “abstract conceptualization” of cultural competence, and links students’ clinical experience with their learning in the formal curriculum. This gradually contributes to one’s “active experimentation” in engaging with cultural diversity, as students are more equipped to make culturally appropriate judgments and demonstrate effective cultural skills. Similar to the four phases of *experiential learning*, the four learning avenues we propose have no starting or endpoint; instead, they form an iterative and cyclical process.

The dynamic process of cultural competence development also conforms with the interactions between *learner agency* and *affordances* in workplace training (Billett & Choy, 2013; Watling et al., 2021). Individuals learn through work as workplaces afford opportunities for learning. These affordances are constituted in routine work practices but are not afforded evenly to all learners (Billett, 2001). The readiness of the workplace to afford opportunities for individuals to engage and access support is key to ensuring the quality of learning (Billett, 2001). Moreover, learner agency, i.e., how individuals elect to engage/participate in social experiences of learning (Watling et al., 2021), shapes their quality of learning. Students, as individual learners, take varying levels of initiative to make active choices and take actions to engage in the clinical environment. The clinical environment affords learners opportunities for learning but can be achieved when individual learners recognize these opportunities and have the capacity to exercise their agency. To maximize their learning, students need to find meaning in the activities and value in what is afforded for them to participate and learn. In turn, students can perceive or construct *affordances* in the workplace setting, as possibilities for sense-making and action take place (Billett & Choy, 2013). The interaction between learner agency and affordances means that individuals’ learning can be dissimilar, which underpins students’ varied learning in clinical placement. This also resonates with previous studies about students’ learning in the informal and hidden curriculum, which can be “opportunistic, idiosyncratic, pop-up, and often unplanned” (Wear & Skillicorn, 2009, p. 452; Winter & Cotton, 2012; Torralba et al., 2020). Therefore, for workplace learning to proceed effectively, how students are afforded opportunities to participate and be supported to engage is key to achieving their personal development.

Grounded in empirical data, our theoretical proposition has the potential to provide clarity about the process of cultural competence development among medical students in clinical placement. Most of the existing theories and models around cultural competence focus on the dynamic, fluid nature of culture, or domains/stages of cultural competence, such as the Cultural Competence Continuum (Cross, 1989), the Purnell Cultural Competence Model (Purnell, 2000), Cultural Humility (Foronda et al., 2016), Cultural Safety (Walker et al., 2009) and Critical Cultural Competence (Almutairi et al., 2015). Very few studies have conceptualized the learning processes of cultural competence.

Our research has tangible implications for cultural competence education in medicine and healthcare. It shows that medical students develop their cultural competence not only through classroom-based formal teaching but also through their engagement in the informal and hidden curriculum. Therefore, being explicit about the informal and hidden curriculum can help students better understand their learning (Neve & Collett, 2018). Our model suggests that a supportive learning environment is essential to foster students’ cultural competence development via the four learning avenues. In the formal curriculum, pre-clinical years can focus on supporting students’ cognitive and affective development through teaching, so they become aware of the importance of cultural competence as a professional requirement. When delivering cultural competence sessions, tutors should signpost potential cultural learning opportunities that students may encounter in the clinical setting. Before embarking on their placements, relevant training is needed to prepare students for their learning in uncertain clinical environments. There is also a need for clinical tutors to incorporate cultural competence as a key learning outcome in students’ placement learning. Continuous support both from the clinical sites and clinical tutors should be in place so that individual learners are afforded ample learning opportunities and prepared to engage in cultural challenges. In addition, reflective activities within a safe environment can be organized for students to discuss and reflect on their cultural encounters in the clinical placement.

Faculty development is key to students’ cultural competence development. A long-term training plan should be put in place to continuously engage medical educators to nurture students’ learning in the clinical setting (Liu et al., 2021). Both cultural competence curriculum and faculty training programs should be guided by research evidence to ensure the contents are meaningful to patients and reflect the needs of society. The authors are strong believers in knowledge co-production (Holmboe, 2017), which incorporates expertise from researchers, educators, clinicians, students, and patients. Therefore, a collaborative team is central to the success of cultural competence education.

This study has several limitations. One limitation is the relatively short period of participant observation in the clinical setting. This was due to practical issues including the length of the chosen clinical block and students’ arranged timetable. Future research may be expanded to explore students’ clinical learning beyond a single clinical block and over a more extended period. Moreover, the majority of participants in this research were Year-1 or Year-2 medical students. It was pragmatic to recruit medical students in the early years of their training because they spent relatively more time on campus and showed a stronger interest to participate in university-wide research activities. Although the authors were able to select more balanced participants via different recruitment strategies, the demographic background of the participants can be further expanded to represent a more diverse cohort of students in medical education. Another limitation of this research is in its transferability to another setting as it is in nature a single-site ethnographic case study. As this research was conducted in one London Medical School with deliberate measures to cultural inclusivity, cultural competence teaching in the school’s formal curriculum and the geographic location of the medical school may have an impact on students’ understanding of cultural diversity and experiences of cultural exposure. Nevertheless, the in-depth qualitative understanding of one medical school with a diverse student body and healthcare environment can provide pedagogic insights to medical schools and identify important indicators to understand medical students’ cultural competence development in general. The results also shed light on understanding medical education in countries or regions that are experiencing an increasing phenomenon of cultural diversity in healthcare.

## Conclusion

This study provides a rare view of how medical students develop their cultural competence in clinical placements, which can inform the development of cultural competence education in medicine and healthcare. The proposed model helps educators gain a holistic perspective of how medical students develop their cultural competence and the essential attributes that are required. The results pave the way for future research to provide details on curriculum structure and pedagogy and explore how students can enhance their cultural competence in different curriculum settings. As this research was conducted in one Medical School, more research is required to provide an in-depth understanding of students’ experiences in different types of medical schools. Moreover, whilst this research acknowledges that students are not a homogenous group, it has not systematically examined the relationship between students’ cultural competence development and their sociocultural backgrounds. How and to what extent their backgrounds may affect their development is beyond the scope of this research and needs further investigation. The strength of this study is that it provides insights to understand the process of medical students’ cultural competence development in the clinical environment, which may also apply to on-placement students undertaking other healthcare programs. The exploratory nature of this study may inform our understanding of healthcare students’ learning experiences around cultural competence in a global context, which makes the study an important addition to the field.

## Appendix 1: AICF Observation template (with sample fieldnotes)

**Table:**

AICF framework Participant: Miss Manda and Lizzy (pseudonymized)	
Date: 01/05/2018	
**ACTIVITIES**	Week Theme: PREGNANCY
**General impressions/Observations**	**Sketch/photo-summary of activities**
I followed Miss Manda and Miss Lizzy (they are clinical partners, one British Chinese and one British Caucasian) for one day. They were allocated to the Early Pregnancy Unit and Emergency Gynaecology Unit this morning. Miss Manda was very early at 8:30 in the ward (she was told to come at 8:30), but the nurse told her to come at 9:00 am in the future. Later, I followed Miss Manda to lots of units in the Early Pregnancy Unit to find her a patient to talk to. I unfortunately did not manage to observe any history taking with Miss Manda. This is because one patient she was asked to talk with was suddenly discharged and the other patient seemed to be angry and refused to talk with any medical students. Fortunately, she was offered a chance to observe the ultrasound scan for a pregnant lady (I was asked to not observe due to privacy and sensitivity issues). I did spend lots of time waiting and talking with Miss Manda and learnt lots of learning experiences on her placements	Scan observation 1: Miss Manda was asked to observe an ultrasonic scan for a pregnancy lady. She told me it was very interesting experience
	Activity 2: Miss Manda was refused a history-checking conversation by a patient
	**ENVIRONMENT**
	Generally, a very busy environment, especially when there is an emergency section in this unit. Usually women had unexpected early-pregnancy symptoms (e.g. miscarriage, sudden bleeding) come here to seek healthcare
Miss Manda did not go to the afternoon session as she said she was not very keen on that	Medical students are often left by themselves to ask and explore. Staff are generally very helpful but too busy. Medical students often feel do not know what to do
	This is a very stressful environment. I saw a few women in pain and crying. In the waiting area, some women are weeping sadly, some with their partners offering support besides. In the waiting area, there is a TV playing health-relevant programmes. I saw one woman was staring at the TV screen with tears coming out of her eyes
**Elements, features and special notes**	
It was a very ‘unhelpful’ morning, according to Miss Manda. She later told me that lots of her peers thought this block was not particularly useful	
I can tell Miss Manda is a very keen student as she is punctual and took the lead in her placement whereas her clinical partner was late by almost forty minutes. She was constantly looking for opportunities to have a history-taking practice with patients	
I can also tell she is a bit nervous on placement. Miss Manda said because she is only Year-2, she doesn’t think she is ‘actually doing anything’ on placements. Year 4 students should be able to help much more	

**Table:**

**INTERACTION**	
**General impressions/observations** (Who is interacting with whom)	**Description of interaction** -medical students interact with patients to learn about communication skills -medical students interact with clinicians to learn about clinical skills, such as how to do an ultrasonic scan -medical students interact with nurses and staff to clarify things and ask for guidance -medical students interact with pair to discuss their case, prepare for their case report, and exchange opinions on a case
Lots of interactions took place between different parties	
Medical students vs. patients Medical students vs. nurses Medical students vs. clinicians Medical students vs. staff Medical students within their pair	
**Elements, features and special notes** Support from peers: Miss Manda says she wouldn’t start talking with patients until her partner is there. This is because 1) she doesn’t want her peer to feel lag behind and 2) she would be more supported if two persons are doing things together to support each other Support from staff: As Miss Manda was scheduled with a patient to talk with to practice history-taking, the nurse-in-charge gave her heads up saying that ‘that is a very angry lady. I’m not sure if she agrees to talk with you’. In fact, it happens that the patient said she was too tired to talk with medical students. I feel this is important information as the head-up prepares inexperienced medical students mentally, so they would not be too sad or self-blaming if knowing this Miss Mandy told me things like this happen, but not very often. Generally, patients are very supportive Unpredictable elements: I agree with Miss Manda that she has not done too much this morning. She was scheduled with another patient to talk with. But the first time she went in, the patient was not there. The second time she went in, the patient went out for a walk. The third time she went in, the patient was discharged already Different institutional contexts: Miss Manda told me that clinical placements experiences in different hospitals and institutions can be very different. For example, some hospitals can be more organised in terms of medical students’ allocation and supervision, some not. This is the same for GP clinics. She told me that some GP placements can be very rigid, such as taking about students’ feedback for the whole afternoon, whereas other GP clinics can be more flexible (some GPs even don’t show up). There are lots of differences in terms of students’ experiences depending on different institutional contexts	

**Table:**

**CULTURAL ELEMENTS**	
**General impressions/Observations**	**Cultural stories heard from others**
Miss Manda told me that she thinks that being culturally competent is extremely important in working in a such diverse place like London. She also mentioned that culture is of different elements, so it is not only what where people come from, but also their educational level, personality, and socioeconomic status Miss Manda shared me with some of her clinical placement experiences last week	Story 1- one patient came to the post-natal ward and does not speak English. They had a Spanish interpreter for the patient via Language Line. Due to bad communication issues, the staff in the hospital did not know that they were expecting an interpreter (ineffective team communication). What is worse, the interpreter was late As the patient is about to deliver a baby, the staff asked around who can speak Spanish. Miss Lizzy tried to help as she speaks Italian and a little bit Spanish but unfortunately medical English in a foreign language is not just easy as a daily conversation. At last, they had to use google translator to help the lady deliver The delivery went OK. The translation applications helped tremendously. But the team were so nervous as being unable to communicate in the same language in a critical medical moment can be very daunting Story 2: Miss Manda told me that she met a doctor who can speak three languages last week. As one patient was unable to speak English, the doctor used Polish to communicate with the patient. This went very well Miss Manda commented that being multilingual is really a great advantage to be a doctor
**Elements, features and special notes** Workforce diversity is a theme for staff. A mixture of staff from different ethnicities work together The patient population is very diverse as well. As I sit in the waiting area, I can hear lots of patients from diverse ethnicities and speak different languages	

**Table:**

**FEELINGS**	
**General impressions/Observations**	**Feelings as an ethnographer**
Diversity is a theme for both the hospital workforce and patient population. Communication challenges (e.g. language differences, national cultural differences) were not noticed during this observation but was shared by Miss Manda Miss Manda encountered angry patient but fortunately she is resilient enough to deal with that	(1) Different learning experiences Depending on where students are allocated and when they are having their placements, they can have very different learning experiences on their placements. Different hospitals and GP clinics can have very different styles in working with medical students. For example, Miss Manda told me that one year-2 medical student was even asked to help with delivery in one of the university’s working hospitals during her placement, though she just started and barely know anything The different learning experiences can also be influenced by other factors such as whether there are consultants and clinicians around to help, and the different cases and personalities of patients (2) Interaction with different parties Lots of parties are involved during students’ placements, such as clinicians, midwives, nurses, lecturers, and peers. This reflects different learning formats including experiential learning, peer learning, and structured classroom learning. To teach cultural competence, Miss Manda said a lecture is a good idea in giving an introduction, but discussion in small groups will help more when it moves to more depth (3) Unconscious learning through exposure/immersion My feeling, also expressed by Miss Manda, is that students are developing cultural competence unconsciously through exposure during clinical placements. But they might not be aware of that. Exposure/immersion is a key factor in developing one’s cultural competence, but the extent of exposure depends on too many elements
**Cultural competence** Knowledge: can be learnt from talking with patients and clinicians. *Afternoon sessions workshop to reflect and exchange cases can enhance the learning experiences.* However, it seems that not all students are keen on this Skills: communication skills are very relevant Miss Manda told me that being culturally competent requires skills to deal with emergencies in critical moments Good example: *When no one is there for language help, the ability to quickly and effectively utilise phone translation software can be of key importance*	

## Appendix 2: Interview question guide

1. How important do you think it is to be culturally competent as a medical student and future doctor?
2. What do you think being culturally competent in the medical setting requires?
3. How do you think the cultural competence teaching at the Medical School has made you aware of the importance to develop cultural competence?
4. How much do you think your medical school recognises the importance of cultural competence education from an institutional level?
5. How culturally competent do you think your tutors (tutors here include academics and clinicians who are involved in teaching in MBBS program) are? Any good and bad examples?
  - Have you identified any culturally competent examples from tutors on placement? If so, how the positive example has impacted your learning?
  - Have you identified any culturally inappropriate examples from tutors on placement? If so, how the negative example has impacted your learning?
6. Where do you think cultural competence has been taught in your curriculum? Any examples?
7. How has your cultural competence been assessed in the curriculum?
8. Can you describe any cultural encounters that you’ve been involved in/observed during students’ clinical placement? (both during the General Practice and hospital rotations)
  - If yes, how do you think these encounters have contributed to your understanding and development of cultural competence?
  - If not, have you heard any cultural encounters in practice from other medical students?
9. How have you been developing your cultural competence on your clinical placements? Any examples?
10. What barriers do you think you have in terms of cultural competence development on placement?
11. What factors do you think can contribute to your cultural competence development on placement?
12. What opportunities do you think you have that can support your cultural competence development on placement?
13. What suggestions do you have to enhance clinical cultural competence education?
14. What do you think the medical school can do to further support students’ cultural competence development?

## Appendix 3: Question guide for focus groups

- Introduction: Can everyone briefly introduce yourselves (name, year of study, region)?
- How important do you think it is to be culturally competent as a medical student?
- What do you think being culturally competent requires in the medical setting?
- In what ways do you think you can develop your cultural competence through exposure on clinical placements?
  Prompts*:*
  o *purposeful observation (patient diversity during history taking and clinical observation)*
  o *unconscious immersion (environmental diversity e.g. ward talks, cultural events)*
- In clinical placements, with whom do you think you can develop your cultural competence by interacting with them?
  Prompts*:*
  o *interaction with culturally competent doctors (role modelling)*
  o *interaction with nurses (learning about working environment, heads-up about patients, some communicational tips)*
  o *interaction with patients and families (history-taking, gaining exposure to diversity)*
  o *interaction with peers (peer support and peer learning, e.g. discussion of cases)*
  o *interaction with other clinical staff (guidance, clarification and heads-up)*
- Whose support in clinical placements do you think is essential in developing your cultural competence?
  Prompts*:*
  o *Staff*
  o *Patients*
  o *Peers*
- In what ways do you think the afternoon workshops in clinical placements can help you develop your cultural competence?
  Prompts*:*
  o *case sharing*
  o *cultural knowledge e.g. children in multilingual environments speak later*
  o *cultural skills e.g. when addressing sensitive questions, frame these as must-ask questions for everyone*
- What environmental elements in clinical placements do you think can influence (both positively and negatively) your cultural competence development?
  Prompts*:*
  o *diversity (patient diversity, workforce diversity, hospital events diversity)*
  o *learning environment (busy, stressful, institutional-different; student-led)*
  o *unpredictability (angry patients, no patients available for practice, no consultants, too many medical students)*
- What individual-level elements do you think can influence (both positively and negatively) your cultural competence development in clinical placements?
  o *e.g. personality, gender, linguistic capability, confidence, personal cultural interest, level of initiatives*
- What challenges have you encountered in dealing with culturally diverse patients on clinical placements?
  o *linguistic challenges*
  o *health literacy-level challenges*
  o *age group challenges (elderly, kids)*
  o *health beliefs challenges*
- How do you think students’ different learning experiences in clinical placements will affect their cultural competence development?
  o *Length of placement*
  o *different learning experiences (e.g. luck)*
- How do you pay attention to developing cultural competence in clinical placement?
  o *active or passive*
  o *conscious or unconscious*
- What do you think the medical school can do to further support students’ cultural competence development on placement?

Note: Italicised prompt points are preliminary results that are used for moderation of the discussion.

## Footnotes

1 The National Health Service (NHS) is the umbrella term for the publicly funded healthcare systems of the UK and consists of primary care (General Practices and community services) and secondary care (hospitals and specialists).

2 The term GP stand for both General Practice (the Surgery) or General Practitioner (Doctors). General Practitioners provide treatment to all common medical conditions and refer patients to hospitals or other medical services for specialist/urgent care. General practitioners in the UK are akin to what might be called family practitioners/physicians or primary care doctors in other health systems.
